# Selecting process quality indicators for the integrated care of vulnerable older adults affected by cognitive impairment or dementia

**DOI:** 10.1186/1472-6963-7-195

**Published:** 2007-11-29

**Authors:** Edeltraut Kröger, André Tourigny, Diane Morin, Lise Côté, Marie-Jeanne Kergoat, Paule Lebel, Line Robichaud, Shirley Imbeault, Solange Proulx, Zohra Benounissa

**Affiliations:** 1 Laval University Geriatrics Research Unit, Hôpital du Saint-Sacrement, Quebec, Canada; 2 Institut national de santé publique du Québec, Quebec, Canada; 3 Centre d'excellence sur le vieillissement, Hôpital du Saint-Sacrement, Quebec, Canada; 4 Faculty of Nursing, Laval University, Quebec, Canada; 5 Direction de la Santé Publique de Québec, Quebec, Canada; 6 Faculty of Medicine and Institut uiversitaire de Gériatrie de Montréal, University of Montreal, Montreal, Canada; 7 Department of Occupational Therapy, Laval University, Quebec, Canada

## Abstract

**Background:**

This study aimed at evaluating face and content validity, feasibility and reliability of process quality indicators developed previously in the United States or other countries. The indicators can be used to evaluate care and services for vulnerable older adults affected by cognitive impairment or dementia within an integrated service system in Quebec, Canada.

**Methods:**

A total of 33 clinical experts from three major urban centres in Quebec formed a panel representing two medical specialties (family medicine, geriatrics) and seven health or social services specialties (nursing, occupational therapy, psychology, neuropsychology, pharmacy, nutrition, social work), from primary or secondary levels of care, including long-term care. A modified version of the RAND^®^/University of California at Los Angeles (UCLA) appropriateness method, a two-round Delphi panel, was used to assess face and content validity of process quality indicators. The appropriateness of indicators was evaluated according to a) agreement of the panel with three criteria, defined as a median rating of 7–9 on a nine-point rating scale, and b) agreement among panellists, judged by the statistical measure of the interpercentile range adjusted for symmetry. Feasibility of quality assessment and reliability of appropriate indicators were then evaluated within a pilot study on 29 patients affected by cognitive impairment or dementia. For measurable indicators the inter-observer reliability was calculated with the Kappa statistic.

**Results:**

Initially, 82 indicators for care of vulnerable older adults with cognitive impairment or dementia were submitted to the panellists. Of those, 72 (88%) were accepted after two rounds. Among 29 patients for whom medical files of the preceding two years were evaluated, 63 (88%) of these indicators were considered applicable at least once, for at least one patient. Only 22 indicators were considered applicable at least once for ten or more out of 29 patients. Four indicators could be measured with the help of a validated questionnaire on patient satisfaction. Inter-observer reliability was moderate (Kappa = 0.57).

**Conclusion:**

A multidisciplinary panel of experts judged a large majority of the initial indicators valid for use in integrated care systems for vulnerable older adults in Quebec, Canada. Most of these indicators can be measured using patient files or patient or caregiver interviews and reliability of assessment from patient-files is moderate.

## Background

Large variations in the quality of health care and services may affect all parts of the population [1]. However, such variations are particularly worrisome for vulnerable older adults [2-4] since for this population increased quality of care is associated with longer survival [5]. Integration of care and service delivery is a promising approach designed to improve access, quality, user satisfaction and efficiency [6-8]. According to a variety of demonstration projects [9-11] the main features of an effective integrated service system for vulnerable older adults are a single point of entry into the system, case management, geriatric assessment and a multidisciplinary care team [12]. The assessment of quality with process quality indicators (PQIs) is considered an essential first step of quality improvement and reduction of its variability [4,13].

This research aims at evaluating the quality of health care and services provided to vulnerable older adults within integrated service systems [14]. The research team selected PQIs for vulnerable older adults affected by cognitive impairment/dementia and being treated in an integrated service system. Indicators were retrieved from published and grey literature in the English language. All of these PQIs were developed outside of Canada. A large number of PQIs came from the United States, where a large-scale research program called the Assessing Care of Vulnerable Elders (ACOVE) project evaluated the quality of care for older adults with the help of PQIs [15,16]. Although the ACOVE researchers developed and validated 236 PQIs for 22 clinical conditions for vulnerable community-dwelling people, aged 65 years and older [17] additional PQIs were considered necessary to assess quality in the context of integrated care.

Since PQIs cannot be transferred between countries without a prior validation, and often translation, to take into account variations in language, culture, and practice, PQIs developed by ACOVE and PQIs from other sources had to be validated before implementation in integrated service systems in Quebec [18]. To our knowledge, previous reports on validation of indicators for transfer between countries [4,18,19] do not specifically consider integrated service systems. We specifically aimed at evaluating face and content validity of PQIs and at assessing measurement feasibility and reliability, in order to obtain PQIs ready for use in the assessment and improvement of quality within regional health administration boards of the public care system in Quebec, Canada. PQIs were not developed for the evaluation of the quality of care of individual providers or specific institutions.

## Methods

### Quality framework

**Figure 1:**
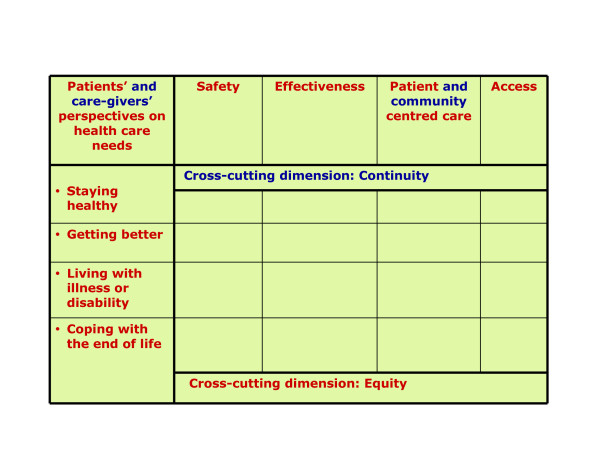
Conceptual framework, adapted and translated from the Institute of Medicine ^25^.

This research on quality assessment for vulnerable older adults in integrated service systems is guided by a conceptual framework to ensure a meaningful and rigorous quality evaluation and improvement [20]. As a preliminary step to the present research we studied existing quality frameworks in the literature and identified the conceptual framework of the Institute of Medicine (IOM) as the most relevant one for our research goals. We modified a published version of this framework, represented by a four-by-four matrix of quality dimensions (safety, effectiveness, patient-centeredness, and access) and patients' perspectives of health care during different life stages (staying healthy, getting better, living with illness or disability, and coping with the end of life) [20]. Within the IOM framework, equity is a cross-cutting dimension of quality, integral to all of its aspects. Equity will be assessed by comparing the quality of care received by different segments of the population, i.e. by stratifying results from indicator assessment according to different characteristics of the study population, such as geographical location, age or gender. Continuity is a quality dimension essential to integrated service systems, especially for vulnerable older adults [7,21]. We therefore added this dimension as a second cross-cutting element to the framework. We also considered the perspectives of the caregivers of vulnerable older patients, along with the patients' perspectives. We enlarged the concept of patient-centeredness to include the patient's community, an important element within an integrated service system for vulnerable older adults (see Figure 1) [14] and translated the resulting framework into French.

### Selecting PQIs

The quality framework was used during the selection process to identify appropriate PQIs for each quality dimension, making sure that all dimensions of the framework could be assessed by PQIs, at least to some extent. Quality can be measured by process or outcome indicators [22]. Process indicators were chosen because they assess the actual care given and its quality [23] and help to detect care and service processes needing improvement. Compared to outcome indicators, process indicators are less influenced by case-mix and other confounding factors [24]. To limit the scope of the present study, we developed a set of PQIs for one clinical condition only. The choice of the condition was based on two criteria: high prevalence among vulnerable older adults and need for integrated services, namely, interventions across the whole care continuum (i.e. ambulatory, short- and long-term care) [25] and from different healthcare disciplines. Cognitive impairment/dementia was chosen because these conditions affect about 40% of adults aged 80 years and older in Canada [26] and require interventions from several kinds of healthcare providers. Some PQIs for other medical problems that frequently affect patients with cognitive impairment/dementia, such as incontinence, pressure ulcers, multiple medications and malnutrition, were also included, such that the final indicator set captures most of the care required by these patients. We selected appropriate PQIs from published and grey literature in the English language and took care to include PQIs for social work and occupational therapy. Selection of indicators was discussed within the multidisciplinary team of researchers from public health, medicine, geriatrics, nursing, occupational therapy, psychology, and pharmacy. We compiled a list of 82 PQIs including 62 ACOVE PQIs applicable to patients affected by cognitive impairment or dementia [27]. Since continuity of care is a specific goal of an integrated service system, the final set of 82 PQIs included 23 PQIs for follow-up care, most of them (21) from ACOVE. A total of 20 indicators came from sources other than ACOVE. These PQIs were included to evaluate care and services like occupational therapy (four PQIs), social work (six PQIs) and pharmaceutical care (two PQIs) [20,28-35]. Non Acove PQIs also cover some aspects of continuity, access and patient-centeredness. Thus the final indicator set should allow evaluating all dimensions of the conceptual framework, the whole care continuum and the full range of services.

### Assessing face and content validity

Two of the authors (SP, EK), having long-time experience with English to French translation of health services related content, translated the 82 indicators into French. A revision by the research team was carried out for all translated material. No back translation method was used since some modifications of the indicators were to be expected during the validation process.

**Figure 2:**
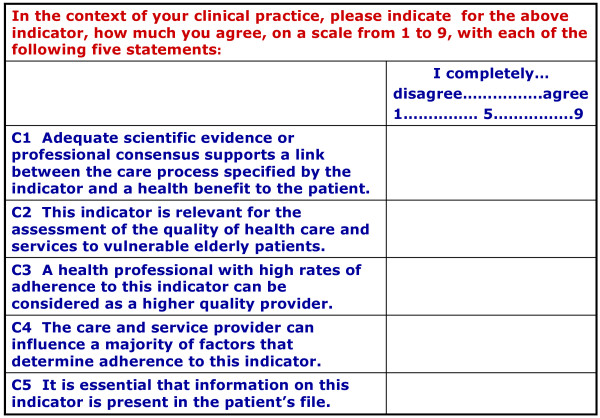
Criteria submitted to the panellists for rating.

The validation process followed a slightly modified version of the RAND^® ^UCLA appropriateness method. This method is a modified Delphi panel, which was privileged since it has successfully been used to develop [17,36] PQIs and to validate PQIs after transfer from another country [4]. PQIs were arranged according to domains of care, namely, clinical evaluation (31 PQIs); treatment (19 PQIs); follow up (23 PQIs); satisfaction, consent and access to care (9 PQIs) (see Appendix, Additional file 1). The panellists rated their agreement with five criteria, namely, (1) scientific evidence for a link between process and outcome, (2) clinical relevance to the care of vulnerable older adults, and (3) ability to discriminate between a high- and a low-quality provider (see Figure 2). The fourth criterion on the provider's influence on factors affecting adherence to the indicator was not included in the final analysis, since a number of panellists reported problems in rating it, due to the interdisciplinary nature of their work. The fifth criterion was about the necessity to document this indicator in the patient's medical record. This criterion was used to guide the assessment of feasibility and inter-observer reliability using the documentation in patients' medical files.

Given the interdisciplinary approach, the panel of 33 members represented nine clinical fields, namely medicine or geriatric medicine (n = 9), nursing (n = 6), occupational therapy (n = 3), psychology (n = 3), neuropsychology (n = 2), pharmacy (n = 4), nutrition (n = 3), and social work (n = 3). The investigators recruited expert practitioners from three major urban centres in Quebec, i.e. Montreal, Sherbrooke and Quebec City, who worked in ambulatory, hospital and long-term care settings. Recruitment criteria were a minimum of five years of clinical experience with a geriatric clientele and ongoing involvement in integrated care for older adults (75 years and older). The PQIs were validated during two rounds of panel consultation where panellists individually rated the PQIs and returned their ratings by mail.

**Figure 3:**
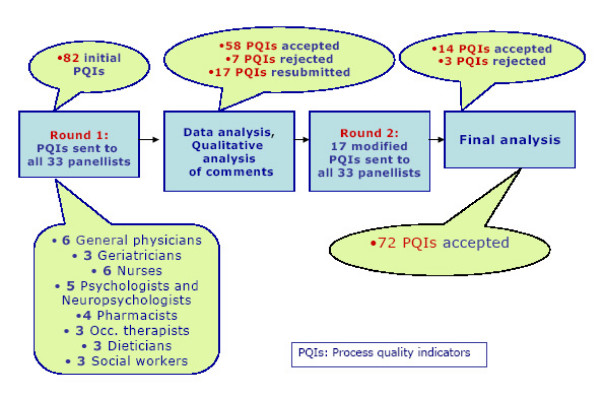
Validation process and results.

The panellists were asked to validate those PQIs that corresponded to their field of expertise, using a form for each PQI. Physicians were asked to validate all 82 PQIs. The forms contained the original English version and the French version of the PQI, its source, the criteria, the judging scale, and a summary of the scientific evidence supporting its use. Panellists indicated their agreement with the five criteria on a rating scale from one to nine, with one signifying complete disagreement and nine complete agreement (see Figure 2). Rating on these scales was later analysed to determine judgment on the PQIs by the panel. Panellists were also invited to comment on, or suggest additional PQIs, and 29 out of 33 panellists did so for 78 out of 82 PQIs after the first round, which helped to modify 23 indicators. A discussion among the research team took place between the two rounds to decide on these modifications. The complete validation process took seven months and is presented in Figure 3.

### Assessing feasibility of measurement and reliability of the selected indicators

**Figure 4:**
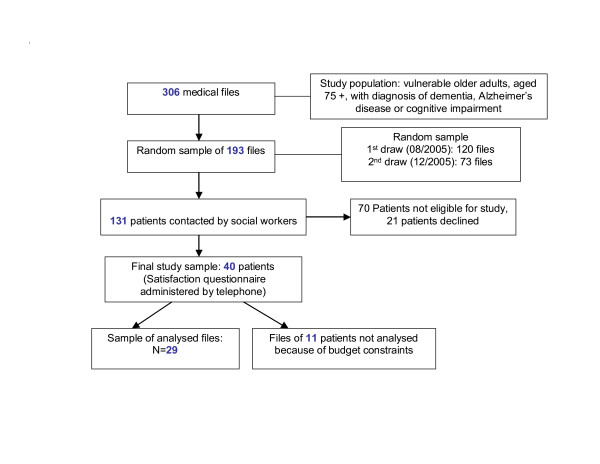
Selection of subjects for the feasibility study.

Measurability and reliability of validated PQIs were assessed in a feasibility study on a random sample of community dwelling patients aged 75 years and older. All eligible patients received home care services from the local health board (*Centre local de services communautaires *(CLSC)) and had a diagnosis of cognitive impairment/dementia (see Figure 4). The Ethics Review Boards of the local Quebec health board, the research centre of the St-Sacrement Hospital and Laval University approved this project. Social workers from the CLSC approached patients or their caregivers and obtained written informed consent for participation. The initial sample of 40 patients or their caregivers completed a telephone interview on patient satisfaction which had been validated in prior research [37]. Four questions were added to this questionnaire to assess four quality indicators not measurable otherwise. Since feasibility of measurement was assessed at the research facility, photocopies of all medical files were obtained for the participating patients. These photocopies came from the CLSC, nine private doctor's offices and four hospitals for the two years prior to recruitment. For each one of the 66 PQIs a form was created to permit abstraction of information from the medical files of the participating patients. Creation of the forms was inspired by a paper published by McGlynn and colleagues [24]. Two study nurses received four days of training on how to abstract information from medical files. Under supervision by the investigators, the nurses then separately went through all medical files for 29 of the 40 patients searching for information regarding each indicator. The nurses were provided a list of those indicators applicable to all participating patients, e.g. indicators E1, E6, E7a (see Appendix, Additional file 1). For each PQI either of the two nurses had to decide whether this PQI was applicable for a particular patient or not, in light of the information retrieved in the file of the patient. If a nurse decided the indicator was applicable for a given patient, she entered information regarding this indicator into the information abstraction form. The nurses did not make a final judgment on whether an applicable indicator was met or not for a particular patient, since the aim of the study was to assess feasibility of measurement only.

### Statistical analyses and judgments

#### Face and content validity

Scores were analyzed after each of the two rounds and comments were used to improve the indicators. Determination of consensus and judgment of indicators followed the RAND^®^/UCLA method. Judgment on a PQI was based on the panellists' rating using the first three out of the five submitted criteria (see Figure 2). The three possible outcomes for the rating results for each indicator were: *appropriate, uncertain *and *inappropriate*.

For an indicator to be judged *appropriate *two requirements had to be met. The first requirement was that panellists agreed with the statement, which meant that the panel's median score was in the upper tertile of the rating scale, or between 7 and 9 (see Figure 2). The second requirement was that panellists agreed among each other. Such agreement between the panellists was assessed with a commonly used continuous statistical measure of dispersion among individual scores, a modified InterPercentile Range (IPR). In the present case the 70% to 30% IPR was used, as suggested by the RAND^® ^working group (personal communication between RAND Europe at the Carlos III Health Institute, Madrid, Spain and the author) [36]. The interpercentile range as a measure of dispersion has been investigated and modified by the RAND^® ^corporation and a version has been developed which applies to any size of panel: it is called the InterPercentile Range Adjusted for Symmetry (IPRAS) [36]. According to the RAND method, if for a given indicator the IPRAS is larger than the IPR, there is agreement among the panellists and if the IPRAS is smaller than the IPR, there is disagreement.

For any given indicator, if the panel's median score was in the lowest tertile of the scale or between 1 and 3, and if there was agreement among panellists, then the indicator was judged *inappropriate*. In all other cases, the indicator was judged *uncertain*.

If an indicator was judged appropriate and modifications were only minor corrections to wording, it was accepted without resubmission. If the modification was major or if the indicator was judged *uncertain*, it was resubmitted for a second round of validation. Indicators judged *inappropriate *were rejected. Final acceptance of an indicator after the second round was limited to those judged *appropriate*. However, if, after the second round, an indicator was judged *uncertain *only by physicians and *appropriate *by the other health care professionals, or vice-versa, an additional statistical measure was calculated. This measure was the rate of acceptance, i.e. the number of all panellists having indicated an agreement with the indicator (score 7 to 9 on the agreement scale), divided by the number of panellists having rated this indicator. If this rate of acceptance was above 80% in the group having judged the indicator as *uncertain*, and the other group had rated it *appropriate*, the indicator was accepted.

#### Inter-observer reliability

Inter-observer agreement was assessed for those PQIs, which were considered applicable and evaluated by both observers (study nurses) at least once for the same patient. A SAS algorithm was created for each indicator according to the requirements for the indicator and regarding the information retrieved from the patient's medical file into the abstraction form. This algorithm allowed to decide whether the indicator was met or not. An overall Kappa statistic was calculated for the agreement between the two observers on whether applicable indicators were met or not.

## Results

All 33 panellists completed the two rounds of validation. Out of the initial 82 indicators submitted to the panel, 72 (88%) were accepted after two rounds of submissions (see Table 1). The rejected indicators concerned all care stages, except evaluation. With respect to the source of the PQIs, 92% coming from ACOVE, 67% from the National Health Service, UK, and 20% from the American Association of Social Workers were accepted. The 12 PQIs from the six other sources were all accepted (see Table 1). The resulting indicator set covered all dimensions of the conceptual framework, i.e., 13 indicators mainly related to security, 31 to effectiveness, 13 to patient-centeredness, 3 to access, and 12 to continuity.

**Table 1:** Results of process quality indicator validation according to source, quality dimension and care domain

Indicator characteristic	Number submitted	Number accepted	(%)
**Source **of indicator			
ACOVE* [27]	62	57	92
AASW* [28, 29]	5	1	20
NASW* [30]	1	1	100
AOTA* [31]	4	4	100
RAND^® ^[32]	3	3	100
Shield and colleagues [33]	2	2	100
Scottish collegiate [34]	1	1	100
NHS* [35]	3	2	67
IOM* [20]	1	1	100
**Quality dimension **covered by indicator			
Safety	13	13	100
Effectiveness	33	32	97
Patient-centeredness	17	13	76
Access	4	3	75
Continuity	15	11	73
**Care domain **to which indicator applies			
Evaluation	31	31	100
Treatment	19	18	95
Follow-up	23	19	83
Consent	2	1	50
Patient satisfaction	3	0	0
Access to care	4	3	75

**All indicators**	**82**	**72**	**88**

All but two out of 72 accepted indicators were measurable either by patient/caregiver interview (four indicators) or from medical files of patients in public (hospital, CLSC) and private (doctor's offices) settings (66 indicators) (see Appendix, Additional file 1). Among 29 out of 40 randomly selected patients affected by cognitive impairment/dementia, information from all medical files over the past two years permitted to assess 63 PQIs in at least one care event for at least one patient. Three PQIs were never applicable among these patients. Four out of the 63 PQIs were found applicable at least once by one observer but were found never applicable by the second. There were 22 PQIs, which could be assessed for at least ten out of the 29 assessed patients, for at least one care event by both observers (see Appendix, Additional file 1). Inter-observer reliability of the judgment whether indicators were met, for all 59 PQIs considered applicable at least once for at least one patient by both observers, was moderate with an overall Kappa-value of 0.57. A telephone interview, which permitted to administer a questionnaire on satisfaction, allowed assessing four of the 72 accepted PQIs for 40 patients. These PQIs covered assessment of access and patient-centeredness.

Since this feasibility study was carried out as a pilot project with limited financial resources, the review of medical files could not be completed for 11 patients. The cost for the review of medical files was 135 Canadian dollars per patient for review by one nurse, including cost for nurse training. Administering the questionnaire on patient satisfaction by telephone was less expensive and cost 49 Canadian dollars per patient.

## Discussion

In this study, panellists judged the face and content validity of a large majority (88%) of the translated PQIs appropriate for use in clinical context within integrated service systems in Quebec, Canada. Measurement of 97% of the accepted indicators is feasible, either by review of photocopies of patient files, performed by study nurses, or via patient's or caregiver's interviews. Reliability can be considered as moderate, given the observed Kappa value, and may be amenable to improvement by increase of training of the study nurses from four to seven days.

In other studies on the transfer of PQIs from one country to another, proportions of accepted indicators varied. Marshall reported that 56.3 % of US RAND^®^/UCLA quality indicators for primary care could be transferred and validated for use in the UK [18]. In a study on the transfer of indicators of preventable drug-related morbidity from the US to the UK, after two Delphi panel rounds, 19 of 57 US indicators (33%) could be transferred and 10 out of 16 new indicators (63%) were accepted, illustrating differences in clinical perspectives and professional attitudes between the two countries [19]. ACOVE indicators have also been transferred from the US to the UK and 86% of PQIs were found suitable for use in England, a result comparable to that of the present study [4]. Careful and thorough development of the ACOVE indicators and a reasonable similarity in the clinical practices of geriatric medicine among the US, the UK and Canada may be the reason for this and for the high level of agreement for PQIs related to safety and effectiveness.

To our knowledge, this is the first report on the transfer, validation and adaptation of PQIs for the care of vulnerable older persons treated within an integrated service system, characterized by a single point of entry, case management, geriatric assessment and a multidisciplinary care team. The multidisciplinary selection and evaluation of face/content validity of the PQIs allowed to take into account the vision of quality of care of all health and social care professions involved in the integrated service system. The resulting indicator set, for which feasibility of measurement has been demonstrated and reliability is moderate, covers all dimensions of the comprehensive conceptual framework for quality guiding this research. Coverage for access is somewhat limited, since only a few PQIs for this dimension could be identified in the literature. To fully explore the dimension of patient- and caregiver-centeredness, satisfaction of patients and caregivers may be assessed as well. To this end, a validated questionnaire on patient satisfaction from prior research was used in the present project [37]. Thus, combining PQIs with the assessment of satisfaction via telephone interview permitted a broad, comprehensive evaluation of the quality of care delivered to vulnerable older adults.

This project is limited to indicators for vulnerable older adults affected by cognitive impairment/dementia. However, efforts are under way in the province of Quebec to validate other PQIs, for example on care for mobility problems. Such efforts should permit to eventually cover all highly prevalent health problems among vulnerable older adults. Also, since the feasibility study was carried out as a pilot project, the number of patients for whom medical files could be assessed was limited to 29. This relatively small number may explain why the number of PQIs, which could be assessed using information from medical files, was limited to 63 out of 72. The restricted number of patients may also be the reason why the number of indicators applicable to more then ten patients out of the 29 was limited to 22 PQIs. Furthermore, several indicators, which theoretically applied to all patients, were not assessed for all patients by both nurses, e.g. PQIs E1, E6 and E7. This discrepancy is reflected in the moderate Kappa value for reliability and suggests improved training of nurses for future research. The information abstraction forms could also be improved using comments from the nurses. A large-scale research project aimed at assessing care of vulnerable older adults with validated PQIs would include a much larger number of patients. Results from such a project may permit to distinguish indicators, which consistently apply to a majority of patients from indicators, which only apply to a small minority. In light of these results, efforts to implement the indicator-set in a continuous quality improvement program may then be limited to the more prevalent indicators. In such a project, the cost per patient for assessment of PQIs may decrease to some extent, given greater training effects for nurses. Once electronic patient files will be available, quality assessment with PQIs via medical data from patients may become economically much more feasible. Finally, one has to caution that a face and content validation of PQIs by clinical experts on the distinction of good quality from bad quality services may not necessarily and easily permit to characterize all aspects of good quality care in the vulnerable older adults' lives.

## Conclusion

This research shows how a set of 72 PQIs for the integrated care of vulnerable older adults can be built by starting out with a quality framework, adapting and then validating PQIs developed elsewhere previously. It further demonstrates that accepted indicators are measurable using medical files of patients from several public and private health care facilities. Finally it shows that the reliability of such PQIs is moderate. PQIs for other highly prevalent health problems affecting vulnerable older adults are presently validated in Quebec and integration of services for these patients is implemented throughout the province. The next step should therefore be a large-scale study assessing care for vulnerable older adults across the province. Thus it will be possible to isolate distinct quality problems in distinct administrative territories. The ultimate goal of this research endeavour is to assess the impact of interventions aimed at continuous quality improvement with the help of PQIs and possibly outcome measures.

## Competing interests

The author(s) declare that they have no competing interests.

## Authors' contributions

EK is a pharmacist and epidemiologist who coordinated most of the phases of this study. She supervised and collaborated in all data analyses and wrote all drafts of this paper. AT is the principal investigator of this research who conceived all parts of this project and contributed to all its stages, including all drafts of the paper. DM is a co-investigator, contributed to all stages and several drafts of the paper and supervised the training of the study nurses and the data collection in the pilot study. LC is a co-investigator and contributed to all stages of the validation process, most significantly to the conceptual framework. M-JK, PL and LR are co-investigators and contributed to the validation process, the planning of the pilot project and drafts of the paper.

SI is a psychologist, contributed to the indicator selection, particularly regarding indicators on social work, to the validation process, the planning of the pilot project and drafts of the paper. SP contributed to the planning of the pilot project, coordinated its data collection and contributed to all drafts of the paper. ZB is a statistician who performed all data analyses for this project and contributed to the paper. All authors read and approved the final manuscript.

## Pre-publication history

The pre-publication history for this paper can be accessed here:



## Supplementary Material

Additional file 1 Appendix. Set of process quality indicators from the ACOVE project and other sources accepted as appropriate and measurable from patients' medical files or by interview. Click here for file
