# Supplementary material for: Selecting process quality indicators for the integrated care of vulnerable older adults affected by cognitive impairment or dementia
Source: BMC Health Serv Res. 2007 Nov 29;7:195. doi: 10.1186/1472-6963-7-195 (PMC2225401; doi:10.1186/1472-6963-7-195)
Supplement: Additional file 1 — Appendix. Set of process quality indicators from the ACOVE project and other sources accepted as appropriate and measurable from patients' medical files or by interview. [file 1472-6963-7-195-S1.pdf]

Appendix 1 **Set of process quality indicators from the ACOVE project and other sources accepted as appropriate and measurable from patients' medical files or by interview**

Process quality indicator	Modified*	Measured by both observers
**Indicators from the ACOVE project**		
**E[†]1)** ALL vulnerable elders newly admitted to a hospital or to a physician practice should receive, within 6 months, the elements of a comprehensive geriatric assessment, including assessment of cognitive ability and functional status [27].	No	23 patients
**E 2)** IF a vulnerable elder is admitted to the hospital for any acute or chronic illness or any surgical procedure, THEN the evaluation should include, within 24 hours, 1) diagnoses, 2) pre-hospital and current medications, and 3) cognitive status [27].	No	9 patients
**E 6)** ALL vulnerable elders should be screened at least once to detect problem drinking and hazardous drinking by taking a history of alcohol use or by using standardized screening questionnaires [27].	No	23 patients
**E 7a)** ALL vulnerable elders should be screened at least once to detect whether they use tobacco regularly [27].	Yes	4 patients
**E 7b)** IF a vulnerable elder uses tobacco regularly, THEN he or she should be offered counselling and/or pharmacological therapy at least once to stop tobacco use [27].	Yes	2 patients
**E 9)** ALL vulnerable elders should receive an assessment of their level of physical activity *at least once a year and, if necessary be provided with counselling about appropriate resources* [27].	After 1st round[‡]	20 patients
**E 10)** ALL vulnerable elders should have documentation that they were asked at least annually about the occurrence of recent falls [27].	No	22 patients
**E 12)** IF a vulnerable elder is admitted to an intensive care unit or a medical or surgical unit of a hospital and cannot reposition himself or herself or has limited ability to do so, THEN risk assessment for pressure ulcers should be done on admission [27].	No	1 patient
**E 13)** ALL vulnerable elders should have documentation of the presence or absence of urinary incontinence during the initial evaluation and annually [27].	No	21 patients
**E 14)** IF a vulnerable elder has a new urinary incontinence that persists for more than 1 month or urinary incontinence at the time of a new evaluation, THEN a clinical conduct based on evidence and including targeted history, physical exam, diagnostic tests and discussion of treatment options should be offered [27].	Adapted	6 patients

Process quality indicator	Modified*	Measured by both observers
**E 15)** IF a vulnerable elder has dementia, THEN he or she should be screened for depression during the initial evaluation [27].	No	17 patients
**E 16)** IF a vulnerable elder presents with new onset of one of the following symptoms: *anxiety, somatic problems*, sad mood, feeling down, insomnia or difficulties with sleep, apathy or loss of interest in pleasurable activities, complaints of memory loss, unexplained weight loss greater than 5% in the past month or 10% over 1 year, or unexplained fatigue or low energy, THEN the patient should be asked about or treated for depression, or referred to a mental health professional within 2 weeks of presentation [27].	After 1st round‡	4 patients
**E 17)** IF a vulnerable elder presents with onset or discovery of one of the following conditions: stroke, myocardial infraction, dementia, malignancy (excluding skin cancer), chronic pain, alcohol or substance abuse or dependence, anxiety disorder, or personality disorder, THEN the patient should be asked about or treated for depression, or referred to a mental health professional within 2 months of diagnosis of the condition [27].	No	3 patients
**E 18)** IF a vulnerable elder receives a diagnosis of a new depression episode, THEN the medical record should document on the day of diagnosis the presence or absence of suicidal ideation and psychosis (consisting of, at minimum, auditory hallucinations or delusions) [27].	No	No patient
**E 19)** IF a vulnerable elder has thoughts of suicide, THEN the medical record should document, on the same date, that the patient either has no immediate plan for suicide or that the patient was referred for evaluation for psychiatric hospitalization [27].	No	No patient
**E 20)** IF a vulnerable elder is being treated for depression, THEN at each treatment visit suicide risk should be documented if he or she had suicidal ideation during a previous visit [27].	No	1 patient
**E 21)** IF a hospitalized vulnerable elder has a definite or suspected diagnosis of delirium, THEN an evaluation for potentially precipitating factors must be undertaken and identified causes treated [27].	No	1 patient
**E 22)** ALL community-dwelling vulnerable elders should be weighed *at least every 6 months* and these weights should be documented in the medical record (*doctor's office, outpatient clinic or CLSC* §) [27].	After 1st round‡	20 patients
**E 23)** IF a community-dwelling vulnerable elder has documented involuntary weight loss (greater than or equal to 10% of body weight) or hypoalbuminemia (< 3.5 g/dl), THEN he or she should receive an evaluation for potentially relevant co morbid conditions, including medications that might be associated	Adapted	2 patients

Process quality indicator	Modified*	Measured by both observers
with decreased appetite (for example, digoxin, fluoxetine, anticholinergics), depressive symptoms, and cognitive impairment and for potentially reversible causes of poor nutritional intake [27].		
**E 24)** IF a vulnerable elder is hospitalized, THEN his or her nutritional status should be documented during the hospitalization by evaluation of oral intake or serum biochemical testing (for example, albumin, prealbumin, or cholesterol) [27].	No	9 patients
**E 25)** IF a vulnerable elder presents with symptoms of dementia, THEN the physician should review the patient's medication list *(prescriptions, over the counter or supplements)* for initiation of medications that might correspond chronologically to the onset of dementia symptoms [27].	After 1st round‡	16 patients
**E 26)** IF a vulnerable elder presents with symptoms of dementia that correspond in time with the initiation of new medications *(prescriptions, over the counter or supplements)* THEN the physician should discontinue or justify the necessity of continuing these medications [27].	After 1st round‡	2 patients
**E 27)** ALL vulnerable elders should be screened for chronic pain during the initial evaluation period and regularly thereafter [27].	No	21 patients
**E 28)** IF a vulnerable elder has a newly reported chronic painful condition, THEN a targeted history and physical examination should be initiated within 1 month and treatment should be offered [27].	No	Never applicable±
**E 29)** IF a vulnerable elder has newly diagnosed dementia, THEN serum levels of vitamin B12 and thyroid-stimulating hormone should be measured [27].	No	5 patients
**E 30)** IF a vulnerable elder has signs of dementia and focal neurological findings that suggest an intracranial process, THEN he or she should be offered neuroimaging (brain computed tomography or magnetic resonance imaging) [27].	No	10 patients
**T†1)** IF a vulnerable elder has newly diagnosed dementia, THEN the diagnosing physician should ask the patient *whether he drives a motor vehicle and, if yes, should inquire at follow-up visits about the patient's capacity to safely continue this activity* [27].	After 1st round‡	5 patients
**T 5)** IF a vulnerable elder is identified as at risk for pressure ulcer development or a pressure ulcer risk score indicates that the person is at risk, THEN a preventive intervention addressing repositioning needs and pressure reduction (or management of tissue loads) must be instituted within 12 hours [27].	No	1 patient
**T 6)** IF a vulnerable elder has mild to moderate Alzheimer disease, THEN the treating physician should discuss treatment with a cholinesterase inhibitor with the patient and the primary caregiver (if available)	No	10 patients

Process quality indicator	Modified*	Measured by both observers
[32].		
**T 7)** IF a vulnerable elder with dementia has cerebrovascular disease, THEN he or she should be offered appropriate prophylaxis against stroke [27].	No	1 patient
**T 8)** ALL vulnerable elders should not be prescribed a medication with strong anticholinergic effects if alternatives are available [27].	No	22 patients
**T 10)** IF a vulnerable elder is prescribed a new drug, THEN the prescribed drug should have a clearly defined indication documented in the record [27].	No	13 patients
**T 11)** IF a vulnerable elder is prescribed a new drug, THEN the patient (or, if incapable, a caregiver) should receive education about the purpose of the drug, how to take it, and the expected side effects or important adverse reactions [27].	No	12 patients
**T 12)** IF a vulnerable elder with chronic pain is treated with opioids, THEN he or she should be offered a bowel regimen, or the medical record should document the potential for constipation or explain why bowel treatment is not needed [27].	No	Never applicable
**T 14)** IF a vulnerable elder with dementia has depression, THEN he or she should be treated for the depression [27].	No	3 patients
**T 15)** IF a vulnerable elder with dementia has a caregiver (and, if capable, the patient assents), THEN the physician should discuss or refer the patient and caregiver for discussion about patient safety, provide education on how to deal with conflicts at home, and inform them about community resources for dementia [27].	No	19 patients
**T 16)** IF a vulnerable elder with dementia is to be physically restrained in the hospital, THEN the target behavioural disturbance or safety issue justifying use of the restraints must be identified to the consenting person (patient or legal guardian) and documented in the chart [27].	No	2 patients
**T 17)** IF a vulnerable elder is physically restrained and the target behavioural disturbance requiring restraint is identified, THEN the health care team should include methods other than physical restraints in the care plan [27].	No	2 patients
**T 18)** IF a vulnerable elder is placed in physical restraints, THEN each of the following measures should be enacted: 1. Consistent release from the restraints at least every 2 hours; 2. Face-to-face reassessment by a physician or nurse at least every 4 hours and before renewal of the restraint order; 3. Observation at least	No	2 patients

Process quality indicator	Modified*	Measured by both observers
every 15 minutes, and more frequently if indicated by the patient's condition, while the patient is in restraints; 4. Interventions every 2 hours (or as indicated by patient's condition or needs) related to nutrition, hydration, personal hygiene, toileting, and range of motion exercises [27].		
**T 19)** IF a vulnerable elder presents with a pressure ulcer, THEN the pressure ulcer should be assessed for location, depth and stage, size, and presence of necrotic tissue, and managed using evidence-based measures (ulcers care guidelines), such as debridement, cleansing, and topical dressings [27].	Adapted	2 patients
**FU[†]1)** IF the elements of a comprehensive geriatric assessment are performed, THEN follow-up should assure the implementation of recommendations [27].	No	13 patients
**FU 3)** IF a vulnerable elder has an advance directive in the outpatient, inpatient, or nursing home medical record or the patient reports the existence of an advance directive in an interview, and the patient receives care in a second venue, THEN 1) the advance directive should be present in the medical record at the second venue or 2) documentation should acknowledge its existence, its contents, and the reason that it is not in the medical record [27].	No	1 patient
**FU 4)** IF a vulnerable elder has specific treatment preferences (for example, a do-not-resuscitate order, no tube feeding, or no hospital transfer) documented in a medical record, THEN these treatment preferences should be followed [27].	No	2 patients
**FU 5)** IF a vulnerable elder enters the hospital, THEN discharge planning should begin *in the days following admission, as soon as the patient's condition stabilizes* [27].	After 1st round[‡]	9 patients
**FU 6)** For ALL vulnerable elders, the *patient's medical record (doctor's office, CLSC[§], hospital and community pharmacy)* should contain an up-to-date medication list [27].	After 1st round[‡]	22 patients
**FU 8)** EVERY new drug that is prescribed to a vulnerable elder on an ongoing basis for a chronic medical condition should have a documentation of the response to therapy *including side effects* [27].	After 1st round[‡]	1 patient
**FU 9)** ALL vulnerable elders should have a drug regimen review at least annually [27].	No	22 patients
**FU 10)** IF a vulnerable elder has been prescribed a cyclooxygenase non selective non steroidal anti-inflammatory drug (NSAID) for the treatment of chronic pain, THEN the medical record should indicate whether he or she has a history of peptic ulcer disease and, if a history is present, justification of NSAID use should be documented [27].	No	No patient
**FU 12)** IF an outpatient vulnerable elder is started on a new prescription medication and he or she has a	After 1st	No patient

Process quality indicator	Modified*	Measured by both observers
follow-up visit with the prescribing physician, THEN the medical record at the follow-up visit should document one of the following: 1) the medication is being taken *and* the physician asked about the medication (for example, side effects or adherence or availability), or 2) the medication was not started because it was not needed or was changed [27].	round	
**FU 14)** IF an outpatient vulnerable elder is referred to a consultant physician, THEN the reason for consultation should be documented in the consultant's note [27].	No	2 patients
**FU 15)** IF an outpatient vulnerable elder is referred to a consultant and subsequently visits the referring physician after the visit with the consultant, THEN the referring physician's follow-up note should document the consultant's recommendations, or the medical record should include the consultant's note, within 6 weeks or at the time of the follow-up visit, whichever is later [27].	No	2 patients
**FU 16)** IF the outpatient medical record documents that a diagnostic test was ordered for a vulnerable elder, THEN the medical record at the follow-up visit should document one of the following: 1) the result of the test, 2) the test was not needed or reason why it will not be performed, or 3) the test is still pending [27].	No	5 patients
**FU 17)** IF a vulnerable elder is discharged from a hospital to home and he or she received a new prescription medication or change in medication (medication termination or change in dosage) before discharge, THEN his medical record *(doctor's office, CLSC[§] or long term care facility)* should acknowledge the medication change within 6 weeks of discharge [27].	After 1st round	5 patients
**FU 19)** IF a vulnerable elder is discharged from a hospital to home or to a nursing home and the hospital medical record specifies a follow-up appointment for a physician visit or a treatment (for example, physical therapy or radiation oncology), THEN the medical record *(doctor's office, CLSC[§] or long term care facility)* should document that the visit or treatment took place or that it was postponed or not needed [27].	After 1st round	4 patients
**FU 21)** IF a vulnerable elder is transferred between emergency departments or between acute care facilities, THEN the medical record at the receiving facility should include medical records from the transferring facility or should acknowledge transfer of such medical records [27].	No	4 patients
**FU 22)** IF a vulnerable elder is discharged from a hospital to home or to a nursing home, THEN there should be a discharge summary in the medical record *(doctor's office, CLSC[§] or long term care facility)*	After 1st round	4 patients

Process quality indicator	Modified*	Measured by both observers
within 6 weeks [27].		
**FU 23)** IF a vulnerable elder is deaf or does not speak English, THEN an interpreter or translated materials should be employed to facilitate communication between the vulnerable elder and the health care provider [27].	No	1 patient
**C[†]1)** IF a vulnerable elder is to have inpatient or outpatient elective surgery, THEN the medical record should document the patient's ability to understand risks, benefits, and consequences of the proposed surgical operation before the operative consent form is presented for signature [27].	No	Never applicable
**Indicators from sources other than the ACOVE project**		
**E 3)** If a patient has early stage dementia, then his performance in productive activities, leisure and everyday activities as well as his ability to drive a car should be evaluated [31].	No	6 patients
**E 4)** If a patient has intermediate stage dementia, then his performance in communicating and personal care should be evaluated [31].	No	7 patients
**E 5)** If a patient has advanced stage dementia, then his performance in swallowing and his positioning should be evaluated [31].	No	3 patients
**E 11)** All care-givers of patients with dementia must be asked about their needs for support services [32].	No	20 patients
**E 31)** All vulnerable elders living in psychosocial circumstances presenting a high-risk for their health should be identified as soon as possible [34].	Adapted	Could not be evaluated within pilot project [#]
**E 32)** If a vulnerable elder is assessed, then language barriers, needs of persons with disabilities (including sensory impairment) or ethnic, cultural and religious preferences should be taken into account [33].	Adapted	Could not be evaluated within pilot project [#]
**T 3)** *If a service plan is necessary, then this plan should be elaborated with the vulnerable elder or his respondent or caregive* [29].	After 1st round	20 patients
**T 4)** If a confidential discussion has to take place with a vulnerable elder, then it should take place in private [33].	Adapted	Assessed in satisfaction interview

Process quality indicator	Modified*	Measured by both observers
**T 9)** No person with dementia should be taking long-acting sedatives (hypnotics, anxiolytics), unless there is an explicit justification for this medication in the medical record [32].	No	19 patients
**T 13)** In order to manage behavioural and psychological aspects of dementia, *no-drug management strategies should always be considered before drug treatment is started* [34].	After 1st round	7 patients
**FU 2)** If a vulnerable elder has symptoms of cognitive impairment but has not received a diagnosis of dementia, then it should be documented that the provider inquired again about those symptoms within 12 months of the first presentation [32].	Adapted	4 patients
**FU 7)** All vulnerable elders with complex medication regimens who are returning to community living *should be evaluated whether they are able to maintain a self-medication program* [31].	After 1st round	10 patients
**A†1)** All vulnerable elders should receive primary care when needed [20].	No	Assessed in satisfaction interview
**A 3)** If a vulnerable older person has to consult a specialist, *then the delay between taking the appointment and the time of the visit should be a maximum of 8 weeks* [35].	Adapted and modified	Assessed in satisfaction interview
**A 4)** If a vulnerable elder needs services or health care at home (nursing care, rehabilitation care, domestic services) then he should receive them, according to a previously established order of priority [35].	Adapted	Assessed in satisfaction interview

* Some indicators were *modified* upon panellists' comments *after the 1st round of* the validation process and some were *adapted* for use in Quebec from the original version found in the literature *before* the validation process.

† Indicators were grouped according to their respective care domain: E 1 to E 30 for evaluation, T1 to T 19 for treatment, FU 1 to FU 23 for follow-up, C1 for consent and A1 to A4 for access.

‡ Text in cursive letters indicates modified part of the indicator.

± "Never applicable" means that none of the two study nurses found this indicator applicable among the 29 patients participating in the study, and thus the indicator was not included in the calculation of the reliability estimate (Kappa value).

§ The *Centre local de services communautaires* (CLSC) is the local Quebec community centre for health and social services.

# The judgment on high-risk psychosocial circumstances appeared too difficult within the pilot project and the diversity of patients in it was too small to evaluate language and cultural barriers.
